# Meta-analysis highlights the key drought responsive genes in genes: *PEPC* and *TaSAG7* are hubs response networks

**DOI:** 10.1186/s43141-022-00395-4

**Published:** 2022-09-02

**Authors:** Sahar Shojaee, Rudabeh Ravash, Behrouz Shiran, Esmaeil Ebrahimie

**Affiliations:** 1 Department of Plant Breeding and Biotechnology, Shahrekord University, Shahr-e Kord, Iran; 2 Department of Plant Breeding and Biotechnology Faculty of Agriculture, Shahrekord University, Shahr-e Kord, Iran; 3 Genomics Research Platform, School of Agriculture, Biomedicine and Environment, Melbourne, La Trobe University, Victoria, 3086 Australia; 4 Australian Centre for Antimicrobial Resistance Ecology, School of Animal and Veterinary Sciences, The University of Adelaide, South Australia, 5371 Australia

**Keywords:** Meta-analysis, Drought, Wheat, Gene expression, Microarray

## Abstract

**Background:**

Wheat is the most important cereal. One of the environmental stresses is drought that harm the production of many cereals and every year due to low rainfall and frequent droughts, the need to produce plants resistant to this stress is felt. Therefore, identification and evaluation of the genes involved in the production of this resistance in plants are of great importance. By identifying these genes and changing their expression, it is possible to produce resistant plants that can tolerate dehydration and drought, with at least a qualitative and quantitative reduction in yield.

**Results:**

Based on the meta-analysis results obtained in this study, in resistant cultivars ~ 4% (2394/61290) of the probe IDs decreased and ~ 4.5% (2670/61290) increased expression, furthermore in susceptible cultivars ~ 7% (4183/61290) of probe IDs decreased and ~ 6% (3591/61290) increased expression (*P* value ≤ 0.05). List of up- and downregulated genes was revealed, among the expressed genes of transcription factors Myb3, ethylene-responsive 5a, MIKC-type MADS-box WM24B, and salinity inducible ERF4 in resistant cultivars and transcription factors WRKY15, MADS-box TaAGL8, WRKY39, and Myb in susceptible cultivars, they showed a significant increase in expression, these transcription factors are of great importance in drought stress. Among them, ethylene responsive 5a in resistant cultivars by 3 times and Myb in susceptible cultivars by 2.6 times have shown the highest expression change. Using Cytoscape Hub software, the *Phosphoenolpyruvate* carboxylase (*PEPC*) and lyase isocitrate (*TaSAG7*) genes, which have significantly different expressions in resistant and susceptible wheat cultivars. *PEPC* and *TaSAG7* genes were upregulated in resistant wheat cultivars as well as down regulated in susceptible cultivars. Also, the qPCR results of selected genes were consistent with the outcomes of the meta-analysis.

**Conclusions:**

All microarray data were collected from the NCBI Gene Expression Omnibus site. Libraries with drought-tolerant and susceptible cultivars for wheat were considered under the stress and control conditions from whole leaf tissue. By meta-analysis combined the purposeful results of multiple experiments, and found list of genes expressed in reverse between the two cultivars. These genes can distinguish between different susceptible and resistant wheat cultivars.

**Supplementary Information:**

The online version contains supplementary material available at 10.1186/s43141-022-00395-4.

## Background

After corn and rice, wheat is the world’s most valuable carbohydrate source for humans [3]. *Triticum aestivum* one of the world’s major crop plants, reducing the yield due to abiotic stresses such as drought and salinity. In dry and semi-dry regions, drought stress is one of the most important environmental factors in reducing the quantity and quality of agricultural. In wheat, as in many other cereals, drought stress is devastating for grain production, with significant negative economic and sociological impacts [5]. Several studies and experiments have been conducted to research and recognize the mechanism of response as well as the tolerance of wheat to these stresses through a pattern of expression of various genes [36].

Drought stress does not occur suddenly in comparison with other tensions and its expansion is gradual so that it intensifies at the end of the period of drought [11]. In the current century, the average ground temperature is predicted to increase by 4.0 ^°^C, which will reduce crop yields by 3 to 4% worldwide [29]. The effect at first can be temporary and may be permanent. Detailed knowledge about the mechanisms of adaptation to drought may be a new approach for the control of plant productivity and survival in drought-prone areas provide [38]. Using omics technology, involved genomics, transcriptomics, proteomics, and metabolomics, can be used to study genomic sequences of the entire genome, transcriptomics of RNA transcripts, proteomics of proteins, and metabolomics metabolites [17].

Microarray data analysis (MDA) purposes to analyze gene expression data obtained using microarray experiments to extract information among genes, across different conditions and different samples. The first being a static set of data simultaneously recording gene expression levels on different samples; the latter registering the evolution of gene expression levels measured on one sample over different time points [13].

The meta-analysis objective is to obtain more information from existing information, which is achieved by overlapping the results of smaller studies and with one or more statistical analyses. Thus, using a meta-analysis of various studies, results that may not be discovered in smaller studies can be achieved. The need to summarize various research has already been taken into consideration. Meta-analysis is a powerful strategy that was designed to overcome the limitations in these gene expression profiling studies. By increasing the statistical power to reveal a more valid The combination of multiple studies enhances the reliability of the results and precise set of differentially expressed genes (DEGs), by integrating same experimental setups and using rank-based tests [52].

A meta-analysis purpose to synthesize information through an explicit statistical protocol of data aggregation and analyses from many individual experimental studies. It is especially effective to answer research questions with broader applicability and uncover emergent properties across individual studies that may not be apparent otherwise. The power of a meta-analysis can be realized when the effects of individual studies are inconsistent in different experimental settings [26].

This study aimed was to investigate the differences in gene expression in resistant and susceptible wheat cultivars in microarray experiments under drought stress with the help of meta-analysis.

## Methods

**Fig. 1:**
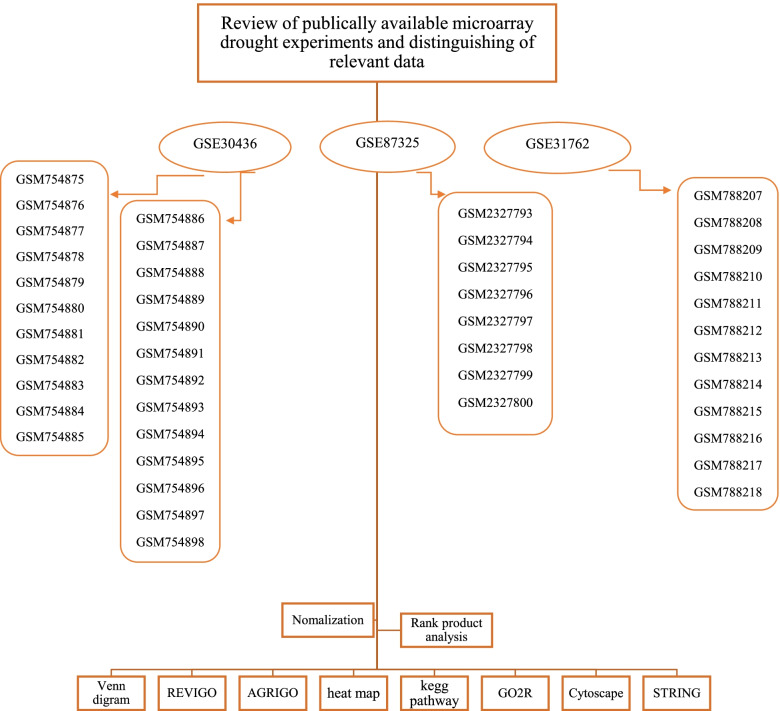
Steps to conduct various surveys on available data for wheat plant

Tasks performed in this study are summarized in Fig. 1.

### Data collection for meta-analysis

Libraries have obtained from Gene Expression Omnibus (http://www.ncbi.nlm.nih.gov/geo) repository. Libraries were downloaded in CEL format. data obtained from total RNA leaves of different varieties of wheat. Drought resistant and drought susceptible cultivars have been distinguished in each library in two conditions of control and stress. (Additional files 1 and 2). The tolerant genotype consists of C306, Bulk, Y12-3, and sensitive cultivars include WL711, Bulk and A24-39. The total number of probe IDs used for meta-analysis were 61290 probs (Table 1).

**Table 1:** Global summary of within species microarray meta-analysis

Plant species	Clade	Studies^a^	Arrays	Probe-sets^b^	DEGs^c^ tolerant	DEGs^c^ sensitive
Wheat	Monocot	3	22	61 k	5 k	5 k

^a^Microarray data research information

^b^Affymetrix Genechip^@^ Microarray of Wheat

^c^number of differentially expressed genes in tolerant and sensitive cultivars

### Microarray meta-analysis

By using the Bioconductor ‘affy’ package, data have normalized in R (http://www.r-project.org) [19]. As well as to reduce the amount of heterogeneity (Batch effects) among studies, the combination of different libraries, modified standards were applied on the data Z [35]. Used the Bioconductor ‘Rank prod’ package to reduce the residual effect of each study species and intensification statistical power to recognize DEGs across experiments [23], which enabled to combine data of different origins and identify DEGs between stress and control conditions. Using the different DEGs with *P* value smaller than 0.05 in response to drought was found in each experiment Found DEG response to drought by Using the different DEGs with *P* value smaller than 0.05 [46].

### Venn diagram

By using the result of meta-analysis, Venn diagram was draw (http://www.interactivenn.net/), from this diagram we have founded similarity of differentially expressed probe IDs under drought stress intolerant and sensitive cultivars.

### Gene ontology (GO) analysis

The DEGs were subjected to the enrichment of gene ontologies (GOs) using the AgriGO toolkit (http://systemsbiology.cau.edu.cn/agriGOv2/). In this site, different DEGs showed in molecular function (MF), biological process (BP), and cellular component (CC) terms [16]. By using the result of AGRIGO, can founded GO term of DEGs down and up intolerant and sensitive individual and P-value for each analysis, got details of categories in REVIGO (http://revigo.irb.hr).

### Gene network

To find the genetic networks, for data extracted from the meta-analysis results, the STRING site (https://string-db.org/) of the ExPASy web site (https://www.expasy.org/), have been used. Name of each gene, converted into its corresponding protein by David site (https://david.ncifcrf.gov/), for use as STRING entry. Protein network of genome-wide functional connectivity is result of assembling all known and predicted protein functional associations for given organism.

### Hub genes

To find important hub genes, correlation between probe IDs normalized expression value was obtained correlation (at 0.95 level), it was performed by Hmisc package in R for finding major hubs we use Cytoscape software. All PPIs in the network were loaded into Cytoscape (v.2.5.0). Used Cyto-Hubba, Plug-in for key genes analysis in network, and find hub genes by a variety of topological study algorithms, namely bottleneck (BN), closeness centrality, clustering coefficient, betweenness centrality, degree, eccentricity, edge percolated component (EPC), maximal clique centrality (MCC), density of maximum neighbourhood component (DMNC), maximum neighbourhood component (MNC), stress centrality, and radiality centrality [8]. To end was conducted using plug-in molecular complex detection (MCODE) in Cytoscape software for extract important gene modules (clusters) with similar expression patterns, module analysis. Following, GO term and pathway enrichment analysis were carried out to probe the biological significance of the detected gene modules.

### KEGG pathways and heat map

The KEGG database is a valuable database for identifying cellular functions [24]. This is accomplished by the procedure of KEGG mapping, especially with the concept of functional orthologs. To generalize the empirical evidence observed in specific organisms and for use in other organisms, molecular network nodes are linked to functional orthologs of the KO database [25]. In this study, due to the unavailability KEGG IDs of wheat plant, DEG genes in susceptible and resistant cultivars separately converted to their homologues in Arabidopsis by using the Plant Ensemble site (https://plants.ensembl.org/biomart). Finally, in KEGG Mapper (https://www.genome.jp/kegg/mapper) KEGG pathway maps were identified.

The heat map of DEGs with filter of having a gene symbol was prepared by the heat map function of the ggplot package.

### Total RNA extraction and qPCR assay

In this study, Australian cultivars of wheat susceptible to drought stress “Sundor” and resistant to drought stress “6o4” and Persian cultivars of wheat, susceptible to drought stress “Tajan”and drought tolerant “Sirvan” were used. These wheat cultivars have been prepared from Karaj Research Center, Iran. Two important genes from meta-analysis results were selected like *phosphoenol pyruvate carboxylase* (*PEPC*) and *lyase isocitrate* (*TaSAG7*) genes. After cultivating these cultivars, drought stress was applied by stopping irrigation in 4-leaf stage. Leaf samples collected in liquid nitrogen separately at three times, 2, 4, and 7 days after cessation of irrigation with the control samples, with two biological replications and two technical repeats. The experiment was performed as a factorial with a completely randomized design. To measure the relative water content (RWC) of the leaves, on the day of the main sampling at 10 AM, a number of leaves from each pot were selected and cut and immediately placed in foil and on ice and quickly transferred to the laboratory. First, their fresh weight was measured and then they were placed in distilled water in refrigerator 4 °C for 24 h to determine their turgid weight. Finally, in order to determine the dry weight, the leaves were placed in an oven at 60 °C for 24 h. The relative water content of the leaves was obtained using the following equation:

$$\mathrm{RWC}\;=\;\frac{FW-DW}{TW-DW}\;\times\;100$$

(DW: dry weight, FW: fresh weight, TW: turgid weight)

RNA extraction from the collected samples was performed using the RNx™-plus kit (Cinagen, RN7713C). The extracted RNAs were purified using DNase. Electrophoresis was performed to determine the quality of the extracted RNA. RNA quantitative test was performed to measure RNA concentration used spectrophotometer. cDNA was synthesized (Yekta Tajhiz Azma, #YT4500, Iran) used a reverse transcriptase enzyme (M-MLV RT) Specific primers were designed by Primer3web version 4.1.0 and then aligned them to find common regions of wheat by NCBI Primer-BLAST. The expression pattern of genes identified in 4 different wheat cultivars under 3 levels of stress (weak, moderate and severe) and control conditions were investigated. The qPCR reaction was performed using YTA Super SYBR Green qPCR MasterMix 2× (Yekta Tajhiz Co., Iran), containing 2× Super SYBR Green qPCR Mastermix and Nuclease-free water and using specific primers for each gene by Rotor-gene Q. *Actin* gene was used as housekeeping gene to normalize. Reactions were performed for each sample with 2 technical replications and two biological replications. Twelve microliters of the reaction mixture was added to each vial (Table 2). Changes in the expression of selected genes were measured using the ΔΔCT comparison method presented by Liwak and Thomas [30]. Analysis of gene expression changes was performed based on a completely randomized design statistical model with General linear model (GLM) and comparison of means with Duncan test (*P* ≤ 0.01) in SPSS software version 22 [47].

**Table 2:** Oligonucleotid primers designed for real-time PCR

Gene name	Gene symbol	Forward sequence (5′–3′)	Reverse sequence (5′–3′)	Product size (bp)	Connection temperature (^°^C)
Phosphoenolpyruvate carboxylase	PEPC	GGTGATCCCTACCTGAAGCA	CTCGTGTCCATCACCTCCTT	152	59
Isocitrate lyase	TaSAG7	AAGGCTCCAGTTCGACATCA	TGCTCCAAGGATGAACTGGT	121	59
Actin		CCTGTGTTGCTGACTGAGGCCCC	CCAAACGAAGGATAGCATGAGGAAGCG	232	59

## Results

### Data collection for different wheat cultivars

The collected data included total RNA of leaf tissues of studied plants. The libraries of the species under study contained 3 susceptible and 3 resistant genotypes. C306 is a cultivar resistant to drought, salinity and heat. C306 cultivar is derived from the cross between the cultivars RGN / CSK3 // 2 * C5 91/3 / C217 / N14 // C281 [21]. WL711 is a semi-short, high yielding, drought-sensitive, medium flowering plant with moderate germination ability. The parents of this figure are [(S308 × Chris) × Kalyansona] [9]. Y12-3 and A24-39 are resistant and sensitive to drought, respectively. Y12-3 is Yehudiyya (35 ° 42′ N; 32 ° 56′ E) which has high water productivity, yield stability and high-water use under drought stress but genotype A24-39 from Amirim (35° 27′ N); 32° 55′ E), which has high efficiency, performance stability and low water use efficiency [28]. The interpretation file for these libraries was obtained from the affymetrix site (https://www.affymetrix.com/), which contains 44 GSM in total.

### Microarray meta-analysis by Rank prod package

In resistant cultivars of the wheat plant, 4% (2394/61290) of DEGs were showed decreased expression and 4.5% (2670/61290) of DEGs, presented increase expression, also revealed in susceptible cultivars that expression of 7% (4183/61290) DEGs were decreased and expression of 6% (2591/61290) DEGs were increased.

### Identify similar and different genes in the DEG by Venn diagram

**Fig. 2:**
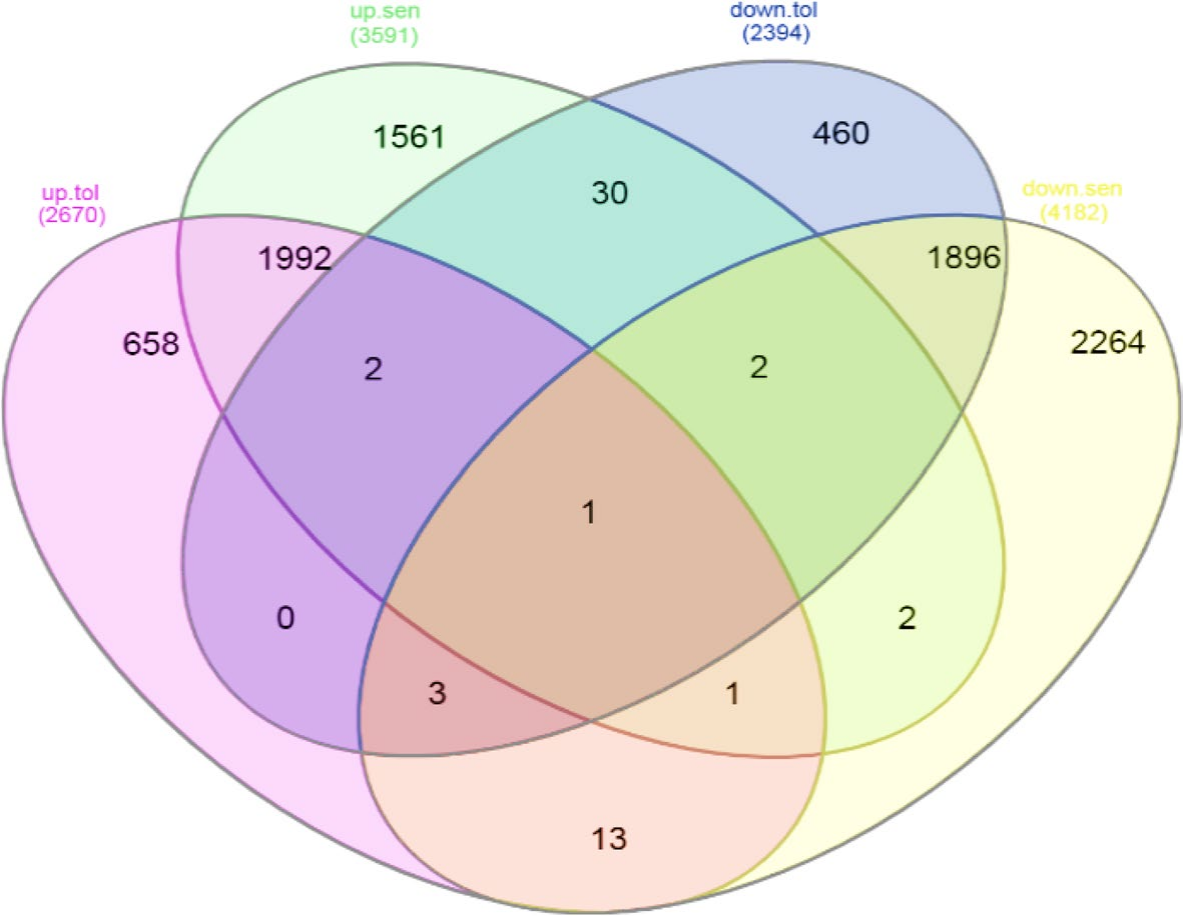
Venn diagram based on wheat significant expression probe ids in result of meta-analysis. 2670 probe ids in tolerant and 3591 probe ids in sensitive cultivar have increase expression. 2394 probe ids in tolerant and 4182 probe ids in sensitive cultivar decrease expression

Venn diagrams were designed on common probes id. Wheat Venn diagram showed that in resistant cultivars were 460 probe ID down expressed and 658 probe ID up expressed. The number of decreased expression probe IDs was 2264 and the number of increased expression probe IDs was 1561 insensitive cultivars (Fig. 2).

Thirteen probe IDs, including phosphoenolpyruvate carboxylase and putative calreticulin, were found to have increased expression in tolerant wheat cultivars, whereas the sensitive cultivars showed a significant decrease (Table 3). Calreticulin (CRT) is an important multifunctional protein that has been identified in many eukaryotic cells except yeast and erythrocytes [33]. At first CRT was known to be an important Ca2+ binding protein, but recent research has shown that CRT plays a role in many cellular functions such as calcium-binding, glycoprotein proper folding, interaction with cellular receptors, RNA binding activity and interaction with the immune response [37]. In higher plants *phosphoenolpyruvate carboxylase (PEPC)* is a cytosolic enzyme and there are several isoforms. *PEPC* is widely distributed in green algae and bacteria, too [45]. Stoma opening, fruit ripening, and seed maturation are different functions for these enzymes. Some of C3 strains have been genetically modified to produce more *PEPC* [34].

**Table 3:** Probe ids that have dissimilar DEGs expression in sensitive and tolerant cultivars

Probe ids	FC^a^	FC^b^	FC^c^	FC^d^	Gene name
TaAffx.121618.1.S1_at	1.34	− 3.62	–	–	–
TaAffx.112692.2.S1_at	1.44	− 2.60	–	–	–
Ta.4208.1.S1_a_at	1.63	− 2.11	–	–	Carboxypeptidase D
TaAffx.12816.1.A1_at	1.47	− 1.91	–	–	–
Ta.27751.2.S1_x_at	1.33	− 1.86	–	–	Chlorophyll a-b binding protein
Ta.5998.1.S1_x_at	2.75	− 1.83	–	–	–
TaAffx.110693.2.S1_at	1.25	− 1.83	–	–	–
TaAffx.93073.1.S1_at	1.29	− 1.83	–	–	–
TaAffx.112708.1.S1_at	1.63	− 1.72	–	–	–
TaAffx.100076.1.S1_x_at	1.56	− 1.64	–	–	–
Ta.6123.1.A1_at	2.72	− 1.60	–	–	–
Ta.26438.1.S1_at	1.12	− 1.58	–	–	–
TaAffx.111711.1.S1_at	1.60	− 1.56	–	–	–
Ta.17782.1.S1_at	5.35	− 1.54	–	–	–
Ta.18480.2.S1_x_at	1.81	− 1.53	–	–	–
TaAffx.100076.1.S1_at	1.54	− 1.49	–	–	–
TaAffx.121565.1.S1_at	1.71	− 1.47	–	–	–
Ta.14106.1.A1_at	2.15	− 1.46	–	–	–
Ta.1156.3.S1_at	1.56	− 1.41	–	–	–
TaAffx.118490.2.S1_at	3.43	− 1.40	–	–	–
Ta.27996.1.S1_at	4.09	− 1.39	–	–	–
TaAffx.112548.1.S1_at	1.56	− 1.36	–	–	–
Ta.6738.1.S1_at	1.44	− 1.30	–	–	–
TaAffx.30914.1.S1_at	2.25	− 1.27	–	–	–
Ta.5314.2.S1_a_at	3.69	− 1.20	–	–	–
TaAffx.59785.1.S1_at	2.82	− 0.93	–	–	–
TaAffx.43047.1.A1_at	1.85	− 0.90	–	–	–
TaAffx.122788.1.S1_at	1.02	− 0.66	–	–	–
Ta.25956.1.A1_at	2.68	− 0.65	–	–	–
TaAffx.53376.1.S1_at	1.57	− 0.61	–	–	–
TaAffx.6656.1.S1_at			1.55	− 1.35	–
Ta.81.1.S1_at	–	–	1.37	− 1.30	Phosphoenolpyruvate carboxylase
TaAffx.25015.1.S1_at	–	–	1.24	− 1.18	–
Ta.27206.1.S1_at	–	–	1.32	− 1.51	–
Ta.23463.2.S1_a_at	–	–	1.14	− 2.44	–
TaAffx.4859.1.S1_at	–	–	1.27	− 1.82	–
TaAffx.56762.1.S1_at	–	–	1.29	− 1.64	–
Ta.14837.3.S1_x_at	–	–	1.46	− 1.20	Putative calreticulin
TaAffx.1266.1.A1_at	–	–	1.38	− 1.26	–
Ta.11507.1.A1_at	–	–	1.22	− 2.08	–
Ta.4964.1.A1_at	–	–	1.91	− 0.87	–
TaAffx.1270.1.S1_at	–	–	1.20	− 1.09	–
Ta.14283.1.S1_x_at	–	–	1.15	− 1.40	–

^a^Fold change of upregulate susceptible wheat cultivars

^b^Fold change of downregulate tolerant wheat cultivars

^c^Fold change of upregulate tolerant, wheat cultivars

^d^Fold change of downregulate sensitive wheat cultivars

Thirty probe IDs such as carboxypeptidase D and chlorophyll a-b binding protein showed significantly high expression in sensitive cultivars but decreased in resistant cultivars (Table 3). In a similar pathway that is the carboxypepteptase-D-arginine-nitric oxide (CPD-Arg-NO) pathway, estrogen, androgen, and *PRL* (prolactin) cause cell survival. CPD membrane-bound metalloproteinase and membrane activity secrete arginine and lysine C-terminal lysine. Further, the CPD acts on the Golgi transport network to process polypeptides/pro-hormones that generate the secretory pathway. CPD exists in plasma membranes [49]. The CPD-plasma membrane acts on extracellular substrates and the arginine are released by CPD transfers to cells that are the common substrates of the two enzymes, arginase, and nitric oxide synthase. Photosynthesis is important for the plant's development, which involves the collection of light and the transfer of solar energy using the chlorophyll a/b light absorption (LHC) proteins. LHC proteins are made from abundant thylakoids encoded by nuclear genes. In higher plants, the LHC protein contains a large 10–12 member gene family and the peripheral light receptors consist of photosystem I (PSI) and photosystem II (PSII) [12], the helices are linked by an ion pair. Each helix binds to chlorophyll molecules (chlorophyll a and b) and some carotenoids in the thylakoid membrane. This type of binding is needed for solar energy transmission, light absorption, and light protection [27].

### Identification different expression transcription factor (TF)

**Fig. 3:**
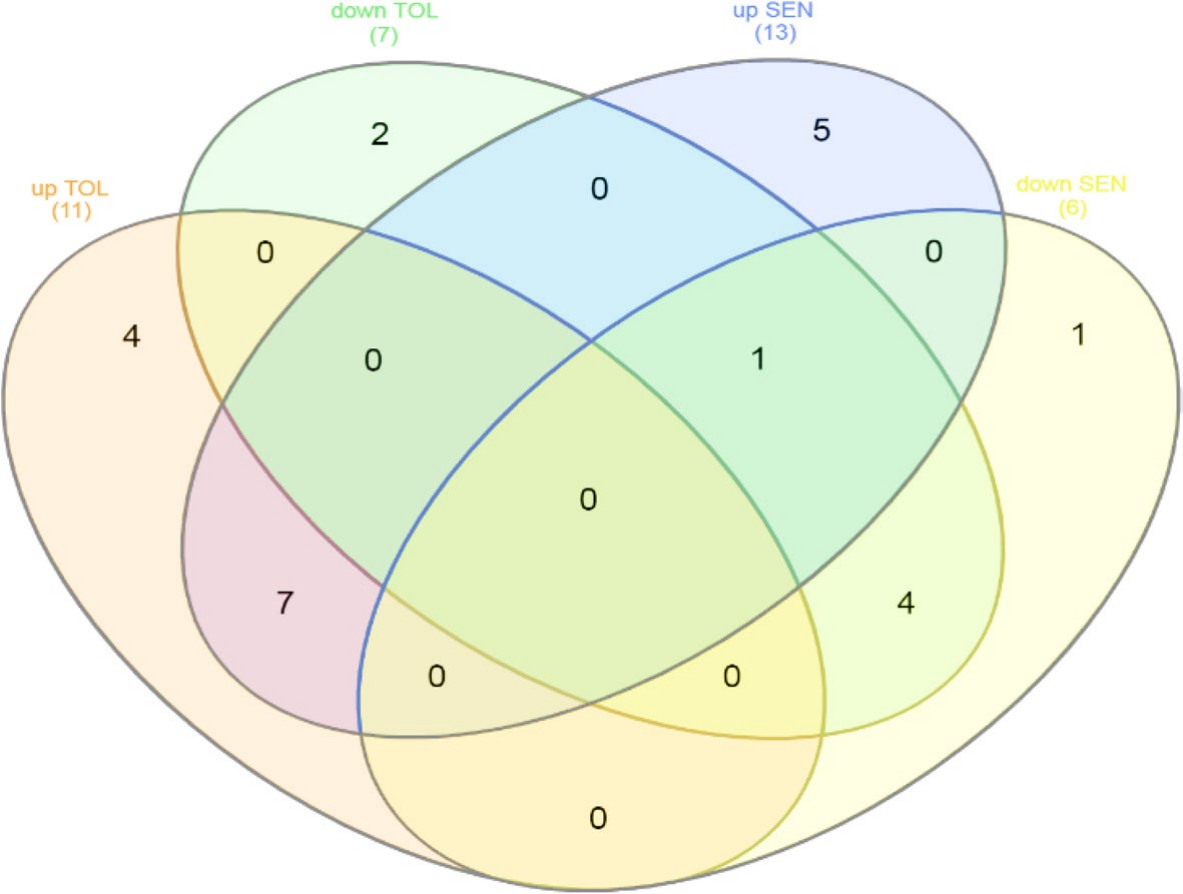
Significant expression Transcription factor genes in result of meta-analysis

Transcription factors play an important role in regulating all processes of a plant’s life [32]. Some types of TFBS elements elaborate in response to different environmental stresses contain MYB, WRKY, HSF, and C2H2 [20]. In this study, 40 transcription factors that identified based on the genes present in the meta-analysis results, showed significant expression changes (Fig. 3). In tolerant and sensitive cultivars, seven transcription factors including MADS-box TaAGL35, drought-responsive factor-like, DRFL1a zinc-finger showed increased expression. Four transcription factors including bZIP, MADS-box TaAGL11, MIKC-type MADS-box, bZip type bZIP1, and WM19A had decreased expression in both tolerant and sensitive cultivars. In resistant cultivars, Myb3, ethylene-responsive 5a, MIKC-type MADS-box WM24B, and salinity inducible ERF4 transcription factors increased expression and two transcription factors, EREBP and NAC NAM, showed a significant decrease in expression. Five transcription factors MYB, WRKY15, MADS-box TaAGL8, WRKY39, increased expression in sensitive cultivars and only one transcription factor, WRKY45, showed a significant decrease in expression. These TFs play different roles in regulating metabolism and plant behavior in different conditions, which is an important reason why they have shown different behavior between susceptible and resistant cultivars.

### Gene ontology characterization in each susceptible and resistant plants

Gene ontology classification of differential expressed genes was performed separately, in susceptible and drought-resistant cultivars. This classification is a common way to interpret transcriptom data as a first step in functional genomic analysis. Study of ontology of expressed genes in three main categories including biological processes, cellular components, and molecular function were classified using REVIGO site as follows.

### Gene ontology refers to the modified genes of susceptible plants

In the molecular function group, expressed genes divided into six categories: glucosyltransferase activity, UDP-glucosyltransferase activity, nutrient reservoir activity, transferase activity, transferring hexosyl groups, transferase activity, transferring glycosyl groups, and transcription factor activity, sequence-specific DNA binding. The highest number of genes was in the category of transcription factor activity and the lowest number of genes was in the category of glucosyltransferase activity.

In biological processes group, expressed altered genes divided into 22 categories. the highest number of genes was in the cellular process category with 150 number in input list and the lowest in the SCF-dependent proteasomal ubiquitin-dependent protein catabolic process, proteasomal protein catabolic process and proteasomal ubiquitin-dependent protein catabolic process with 5. The cellular group includes the smallest category. The expressed genes in this subgroup are divided into two categories. The ubiquitin ligase complex and SCF are ubiquitin ligase complex.

### Gene ontology related to the modified genes of resistant plants

The gene ontology of altered genes expressed in resistant cultivars was divided into two groups of molecular function and biological process. In the biological subgroup, the altered genes were in the response to stress category and in the molecular subgroup, the genes were in the nutrient reservoir activity category.

### Gene network of drought resistance

**Fig. 4:**
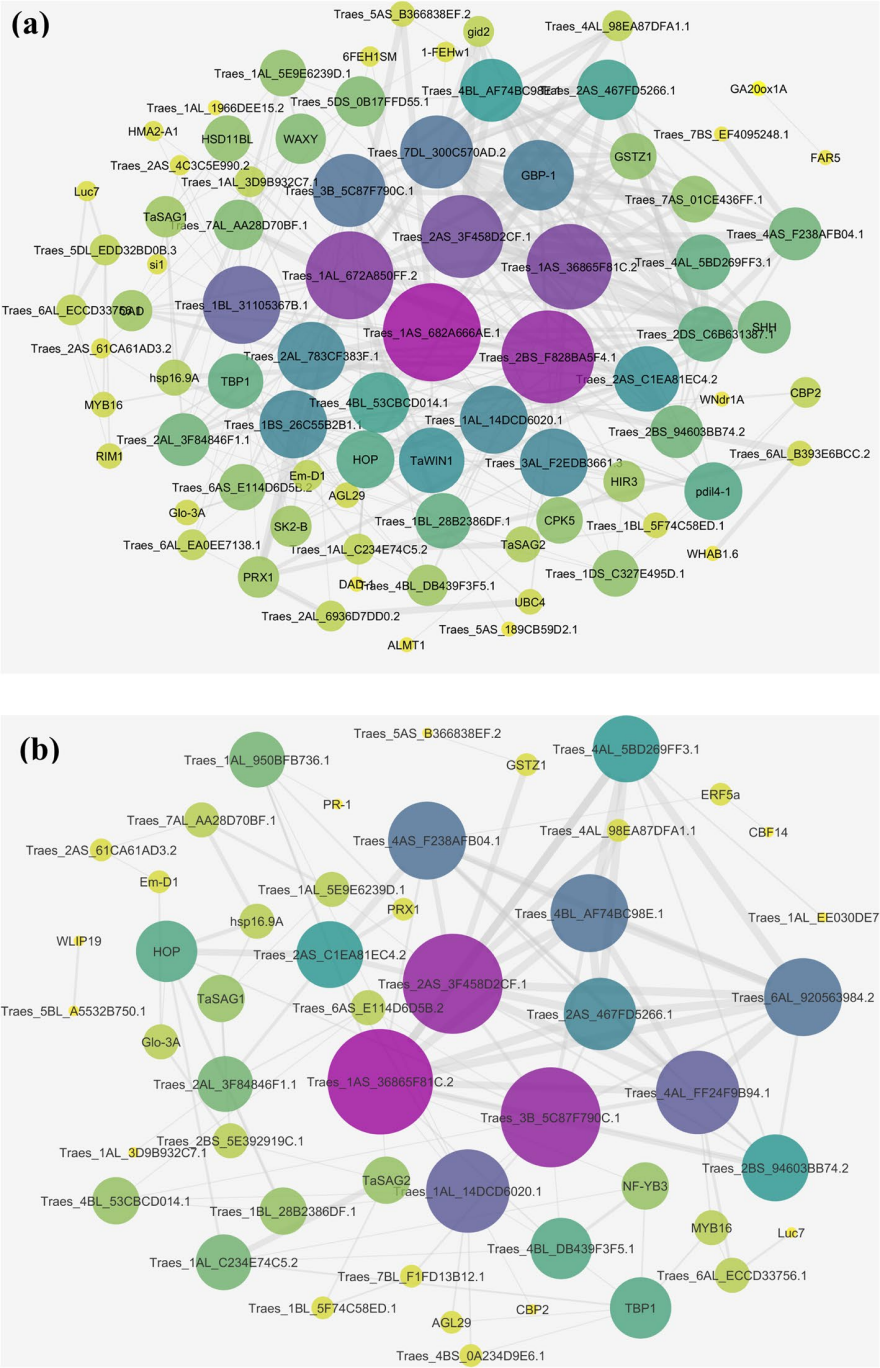
Gene network for the wheat, sensitive cultivars (**a**) and tolerant cultivars (**b**). If the line is not connected to the protein, it does not indicate the association between that protein and other proteins. The number of lines further indicates that there is a large association between that protein and other existing proteins

Network structure and subnet analysis were done, according to the Protein-protein interaction (PPI) dataset downloaded from STRING. The resulting PPI network was 180 proteins have been investigated, 106 proteins are related to proteins of expressed genes in sensitive wheat cultivars and 74 proteins are related to proteins of resistant cultivar genes. The giant component which included the majority of the entire network protein containing 74 nodes in resistant cultivars and 106 nodes in sensitive cultivars (Fig. 4).

### Hub genes of drought resistance: PEPC and TaSAG7

**Fig. 5:**
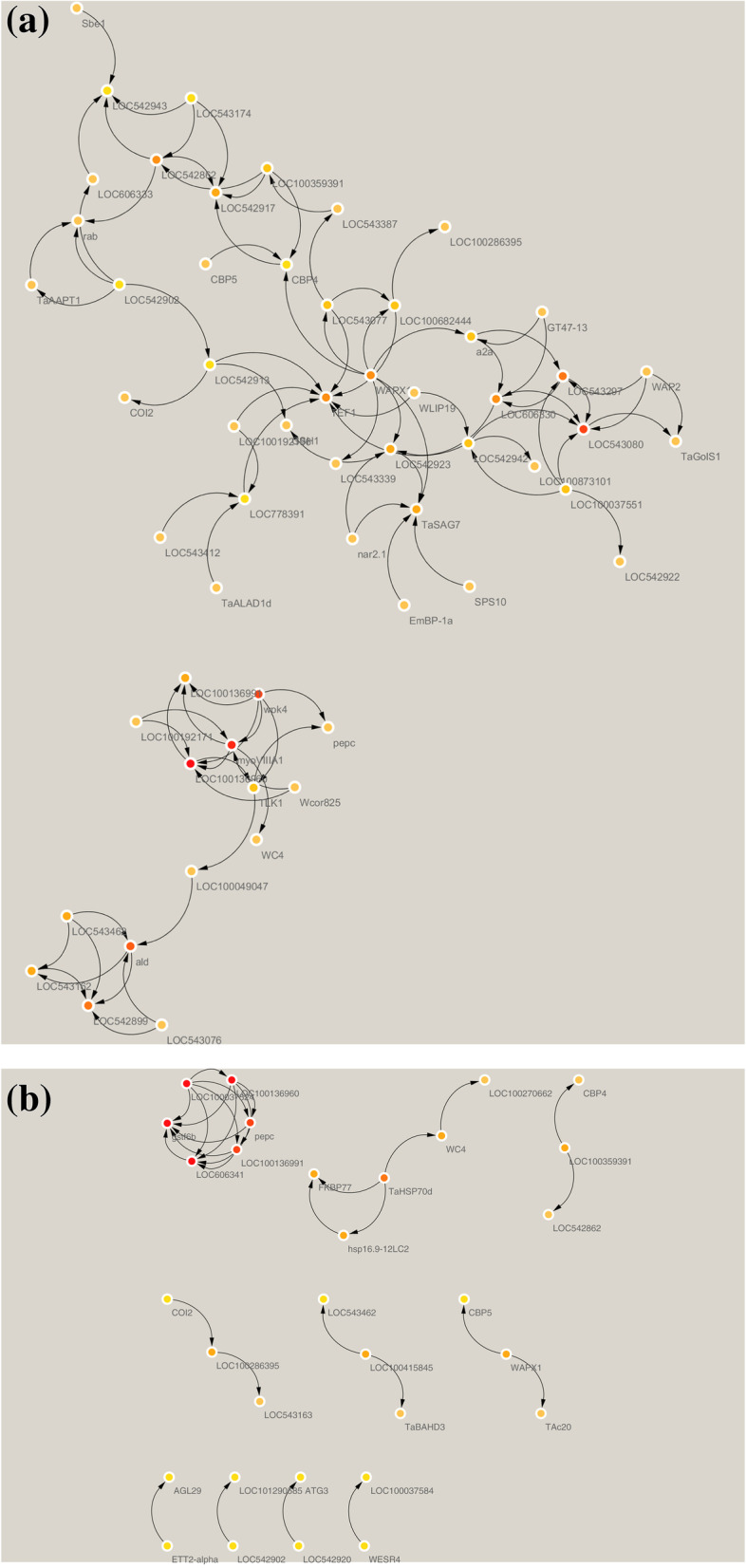
Cytoscope diagram based on the symbols gene. **A** Resistant cultivars. **B** Sensitive cultivars The ranking is based on the MCC method in cytoHubba. Red to yellow, respectively, indicates a higher to lower rank

Cytoscape software has been used to identify effective genes among meta-analysis datasets. It is a popular platform for analyzing biological networks [41]. Twenty-five hub genes, using the MCC method based on the correlation of gene expression limited to probes that have gene names from the meta-analysis results, have been identified for sensitive and tolerant cultivars individually. The results showed that in sensitive cultivars of ribosomal protein P1, glutathione transporter, SPP2 and also for tolerant varieties of vascular pyrophosphate H+, myo protein VIIIA1, glucose transporter protein and wpk4 protein kinase were highly ranked (Fig. 5) (Tables 4 and 5). The RPP1 ribosomal protein is one of three phosphoproteins in the large 60S subunit of the eukaryotic ribosome. RPLP1 plays an important role in the long-term phase of protein synthesis. RPLP1 provides a heterodimer with RPLP2 dimers [7]. The glutathione transferase gene (GSTs; also known as glutathione S-transferase) are the major stage II detoxification enzymes mainly found in the cytosol. In addition to the role of enzymes in catalyzing the binding of electrophilic substrates to glutathione (GSH), they also perform other functions. They have peroxidase and isomerase activities, which can inhibit N-terminal June kinase (thus cells protected from H2O2-induced cell death) [40]. Sucrose is an essential carbohydrate for plants and other photosynthetic organisms and is known as one of the major photosynthetic products. The SPP2 gene catalyzes sucrose biosynthesis in the final step. SPP encoding genes have been described in various plant species including Arabidopsis, tomato, rice, wheat, corn, and coffee. Four genes show homology to SPP in Arabidopsis, whereas three and four genes are described in wheat and rice, respectively [1]. Blastn analysis by NCBI site was performed for the two selection genes in rice and Arabidopsis plants. The results show *PEPC* sequence in Arabidopsis has 95.12% identity sequence and Accession is X98080. *TaSAG7* sequence in Arabidopsis has 72.62% identity sequence and Accession is AK316978. *PEPC* sequence in rice has 84.48% identity sequence and Accession is CP056060. *TaSAG7* sequence in rice has 88.76% identity sequence and Accession is XM_015789456.

**Table 4:** Hub genes identified for susceptible varieties

Rank	Gene hub for susceptible varieties	Name gene	Score^1^
1	LOC606341	Ribosomal protein P1	48
1	gstf6b	Glutathione transferase	48
1	LOC100037524	Sucrose-6F-phosphate phosphohydrolase SPP2	48
1	LOC100136960	Vacuolar H+-pyrophosphatase	48
5	pepc	Phosphoenolpyruvate carboxylase	24
5	LOC100136991	GTP cyclohydrolase 1 isoform TaC	24
7	TaHSP70d	HSP70	3
8	LOC100286395	Putative GA receptor GID1	2
8	LOC100415845	Aci-reductone-dioxygenase-like protein	2
8	hsp16.9-12LC2	Heat shock protein 16.9	2
8	WAPX1	Ascorbate peroxidase	2
8	FKBP77	Peptidylprolyl isomerase	2
8	LOC100359391	ATP synthase subunit	2
8	WC4	Cysteine proteinase inhibitor	2
15	COI2	Coronatine insensitive 2-like protein	1
15	ETT2-alpha	ETTIN-like auxin response factor	1
15	AGL29	MADS-box transcription factor TaAGL29	1
15	LOC543462	20S proteasome beta 7 subunit	1
15	LOC542902	Catalase isozyme	1
15	LOC101290585	Fatty acid alpha-dioxygenase	1
15	LOC542920	Glutathione peroxidase-like protein	1
15	ATG3	ATG3 protein	1
15	CBP5	Chlorophyll a-b binding protein	1
15	WESR4	Zinc-finger motif	1
15	LOC100037584	Fasciclin-like protein FLA25	1

^1^Scores are based on the MCC method

**Table 5:** Hub genes identified for resistant varieties

Rank	Gene hub for resistant varieties	Name gene	^a^Score
1	LOC100136960	Vacuolar H+-pyrophosphatase	12
2	myoVIIIA1	MyoVIIIA1 protein	11
3	LOC543080	Sugar transporter protein	10
3	wpk4	wpk4 protein kinase	10
5	Ald	Ald protein	9
6	LOC542899	QM	8
6	LOC543297	Cathepsin B	8
8	LOC542862	Alpha 1,4-glucan phosphorylase	7
8	LOC606330	Calreticulin-like protein	7
8	WAPX1	Ascorbate peroxidase	7
8	TEF1	Translation elongation factor 1 alpha-subunit	7
12	LOC100136991	GTP cyclohydrolase 1 isoform TaC	6
12	LOC542923	Elongation factor	6
12	LOC542917	26S proteasome ATPase subunit	6
12	LOC543462	20S proteasome beta 7 subunits	6
12	LOC543162	TOM7-like protein	6
12	TaSAG7	Isocitrate lyase	6
18	LOC542942	Ferredoxin	5
18	LOC543077	Uncharacterized LOC543077	5
18	LOC100359391	ATP synthase subunit	5
18	LOC100682444	NF-YB3	5
18	a2a	Glycosyltransferase	5
18	TLK1	Tousled-like protein kinase	5
18	LOC100037551	Triticain alpha	5
25	LOC543174	Cytoplasmatic ribosomal protein S13	4

^a^Scores are based on the MCC method

### KEGG pathways and heat maps

**Fig. 6:**
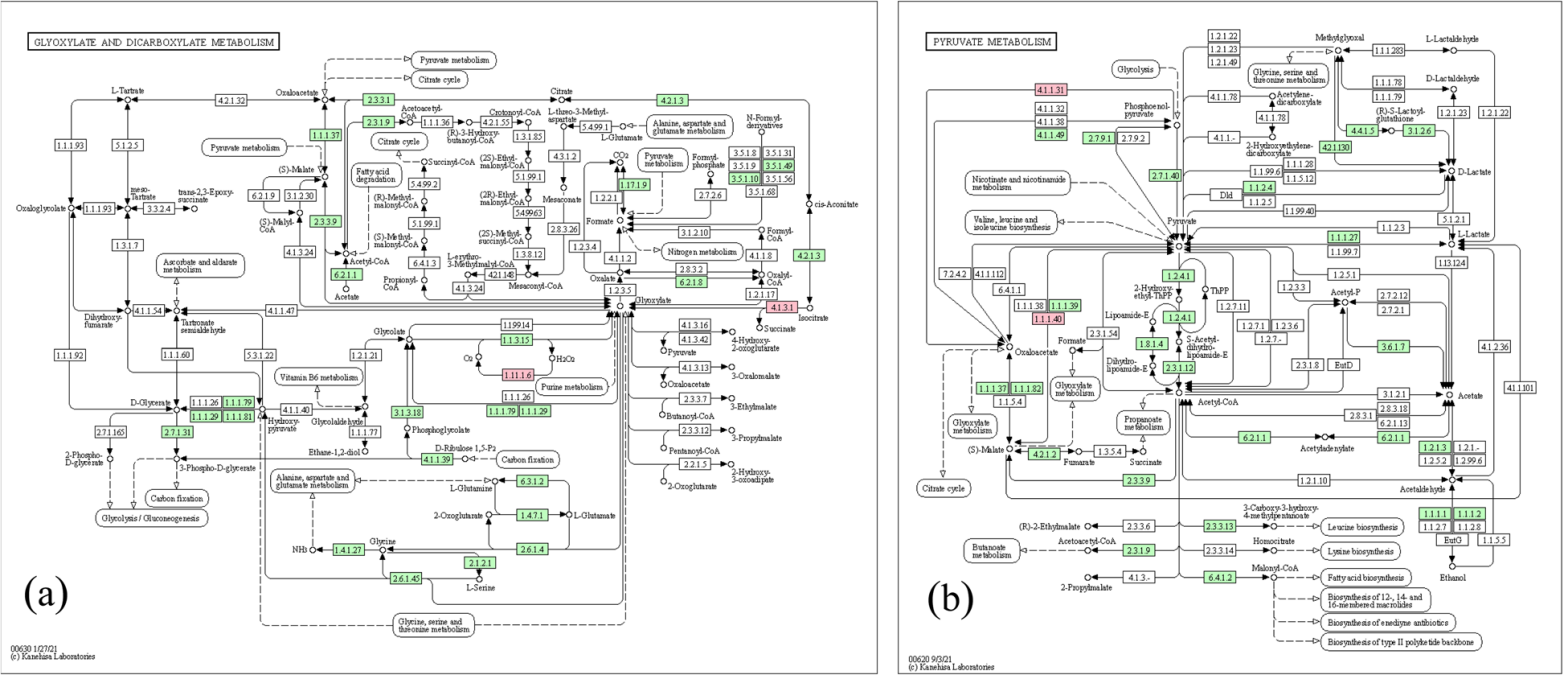
KEGG pathway maps of *glyoxylate and dicarboxylate metabolism and pyruvate metabolism*. *TaSAG7* and *PEPC* genes are active in these two pathways, which are important genes in drought tolerance

**Fig. 7:**
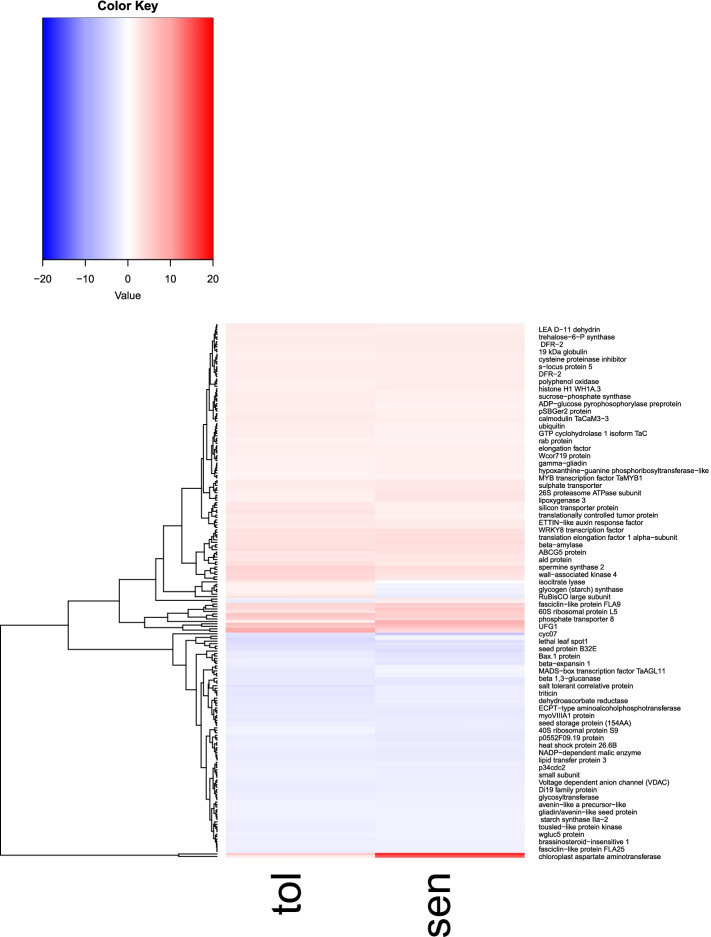
Heat map of DEGs in the resistant and susceptible wheat cultivars. The red bars indicate increases and the blue bars indicate decrease expression genes

Isocitrate lyase (ICL) plays an important role in the metabolic processes of citric, methylcitric, and glyoxylate cycles [39], which is the bypassed pathway of the TCA cycle that converts isocitrate to glyoxylate and succinate. During germination, ICL plays a key role in lipid-sugar conversion using the acetyl unit from acetyl-CoA in arabidopsis, the product of β-oxidation, by the glyoxylate cycle and gluconeogenesis [10] ICL is a single-copy gene in both rice [31] and Arabidopsis [50]. ICL and malate synthase were involved in the transfer of leaf peroxisomes to glycosystems and this process was correlated with aging and senescence [55]. Phosphvanol pyruvate carboxylase (*PEPC*) is a cytosolic enzyme in higher plants and is also widely distributed in green algae and bacteria. In higher plants, there are several PEPC isoforms. These enzymes are involved in a variety of functions, including stomatal opening, fruit ripening, and grain maturity. To date, several C3 species have been genetically engineered to produce more PEPC [42]. The KEGG pathway identified for *isocitrate lyase* and *PEPC* genes have played an important role in resistance to drought stress in wheat (Fig. 6). The expression heat map of the identified genes is shown expression difference in susceptible and resistant gene profiles [54]. (Fig. 7, Table 3).

### Laboratory studies of PEPC and TaSAG7 genes by qPCR

**Fig. 8:**
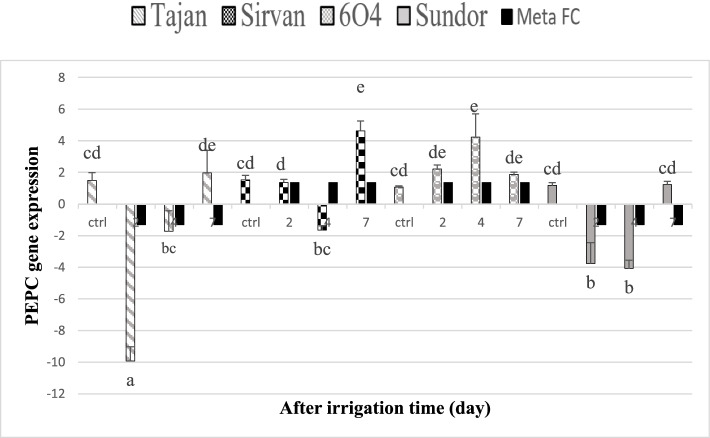
Expression level of *PEPC* gene in tolerant wheat (6O4 and Sirvan) and sensitive wheat (Sundor and Tajan) genotypes at three stress levels 2, 4, and 7 days after drought stress. FC column based on the results of meta-analysis performed for susceptible and resistant cultivars placed separately next to each expression level columns for better comparison of the results

**Fig. 9:**
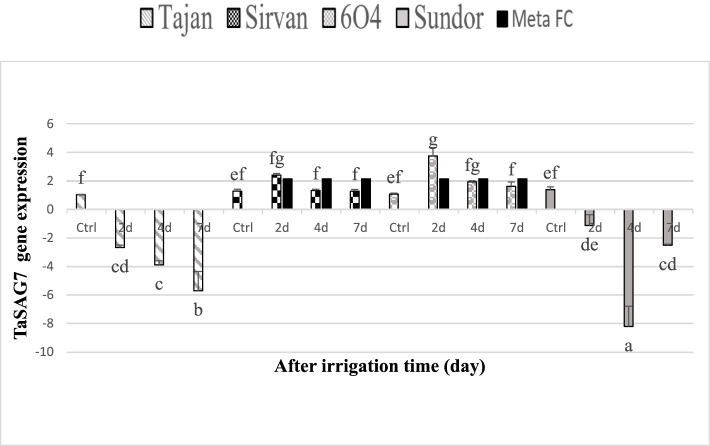
Expression level of *TaSAG7* gene in tolerant wheat (6O4 and Sirvan) and Sensitive wheat (Sundor and Tajan) genotypes at three stress levels 2, 4, and 7 days after drought stress. Based on the results of meta-analysis, this gene has increased only in resistant cultivars. For convenience of comparison, the FC column obtained from the meta-analysis placed next to the resistant cultivars

**Fig. 10:**
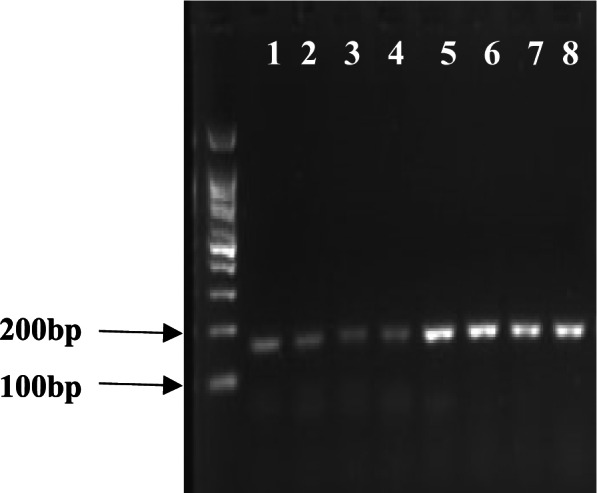
Real-time-PCR (RT-PCR) of *PEPC* gene under normal conditions and drought stress. Bands 1–4 show RT-PCR under the normal conditions and bands 5–8 show RT-PCR under drought stress

**Fig. 11:**
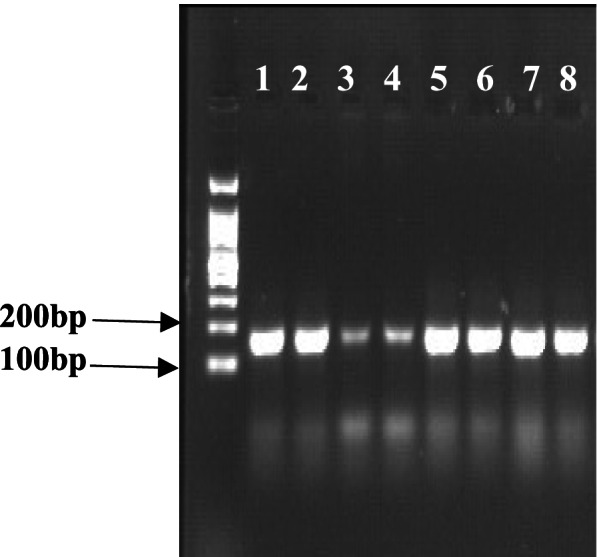
Real-time-PCR (RT-PCR) of PEPC gene under normal conditions and drought stress. Bands1–2 and 5–6 show RT-PCR for the normal conditions, while bands 3–4 and 7–8 show RT-PCR under drought stress

We used qPCR to investigate the expression change of two selected genes at four-point time, control, 2, 4, and 7 days after cessation of irrigation in leaf tissue of different wheat cultivars (Figs. 8 and 9). The amount of RWC for 2 days of stress decreased for two tolerant cultivars 6O4, 3% and Sirvan 13% and also for Sundor cultivar 2% and Tajan 15%. The amount of RWC for 4 days of stress decreased for two tolerant cultivars 6O4, 15% and Sirvan 31% and also for susceptible cultivars Sundor 25% and Tajan 24% and finally the amount of RWC for 7 days of stress decreased for two tolerant cultivars 6O4, 48% and Sirvan 48% and also for susceptible cultivars Sundor 46% and Tajan 52%. Numerous studies have reported the negative effects of increased *PEPC* activity on Pn or biomass. For example, production of C4 *PEPC* in rice led to a decrease in photosynthesis due to increased respiration under light conditions and severely reduced growth [43]. Other studies have shown that transgenic plants expressing *PEPC* have relatively high biomass under stress conditions such as optical oxidation, heat, and drought. C4 *PEPC* has been cloned and identified in many crops including rice, wheat and Arabidopsis. Expression of maize *PEPC* gene in transgenic rice plant has increased antioxidant capacity under drought stress [42]. *PEPC* maize gene expression in transgenic rice plant increases antioxidant capacity under stress conditions such as light oxidation, heat, and drought [15]. In general, based on the meta-analysis, *PEPC* gene increased expression by 1.37 in resistant cultivars and decreased by − 1.30 in susceptible cultivars. The expression of this gene was expected to increase in Sirvan and 6O4 cultivars and in two cultivars. Tajan and Sundor dropped. By examining the mean comparisons made in this study, the results obtained from qPCR were consistent with the results of meta-analysis (Figs. 10 and 11).

During biotic and abiotic stresses, the activity of plant defense systems against free radicals decreases and the production of oxygen free radicals increases. This destroys cell membranes through peroxidation of phospholipids and stops intracellular activities, especially enzymatic reactions. Providing a carbon skeleton for carbohydrate synthesis is not the only role of the glycoxylate cycle. This pathway has also been shown to play an anaplastic role in microorganisms and plants. The glyoxylate cycle can play this vital role through the net production of succinate from acetyl coa [2]. The two enzymes lyase isolate and malate synthetase are unique to the glyoxylate cycle, which avoids the decarboxylation steps of the Krebs cycle. Based on a meta-analysis of TaSAG7 gene in resistant cultivars, a 2.14-fold increase in expression has been reported, which results from real-time PCR are consistent with the results of meta-analysis and confirm the result of meta-analysis.

## Discussion

Due to climate change, greenhouse effects and lack of available resources, as well as successive droughts, research is needed to develop plants that are resistant to these changes. Drought is a complex trait that sometimes affects crop yields. Drought tolerance is a complex trait, make an impact on many genes and mostly conditioned by many component responses, which may interact and may be different with respect to types, intensity and duration of water deficit [22]. In this study, libraries containing microarray data of wheat plant were used for several of drought sensitive and tolerant cultivars. Based on the comparison of meta-analysis between resistant and susceptible cultivars, a total of 6261 probes increased expression and 6576 probes decreased expression. By identifying the genes of each probe, common and different genes were identified in each category. By examining protein-protein interactions, important nodes were identified, including protein kinases that induce mitotic divisions, heat shock proteins, proline 5-carboxylase synthase, ribosomal proteins, and ubiquitin. Creating plants resistant to drought stress are effective. Transcription factors identified include WRKY, MADS-box, and bZIP. Transcription factors are proteins that specifically bind to activators around the gene or earlier regions and depending on its type, gene expression is reduced or reversed after binding of these factors [4]. These factors affect RNA polymerase and regulate gene expression by inhibiting or stimulating its activity. The number of transcription factors in different organisms varies and depends on the size of the existing genome, the larger the genome, the greater the number of transcription factors [51]. Family name of the WRKY is among the largest transcriptional regulator families in plants and participate as activators and inhibitors in important plant processes [44]. These transcription factors are regulators that have both positive and negative activities. WRKY is one of the ten largest gene families found in high plants and all green plant ancestors. During this evolution, genes of this family have adapted to the complexities of the pathogenic defense mechanisms. In this research transcription factor WRKY15 and WRKY39 in susceptible cultivars increased expression and only one transcription factor called WRKY45 showed significant decrease in expression. MADS-box transcription factor plays an important role in developing lateral root, determining meristem type, and especially flower formation. The MADS-box motif is a conserved region of 56 amino acids, which has been observed in the DNA binding domain of many eukaryotic transcription factors. The most prominent features of the MADS-box gene family are the diverse functions of its members that affect different aspects of plant growth and development. Among different families of eukaryotic transcription factors, the basic leucine zipper (bZIP) family is one of the largest and most diverse families. Members of this family are involved in various processes including response to biological or non-biological stimuli, seed maturation, embryogenesis, and transduction pathways responsible for attacking pathogens, flower development, and vascular systems. This family is characterized by the alkaline region required for DNA binding and the zipper region of leucine required for dimerization. Members of this family have been studied in various plants such as Arabidopsis, rice, maize, grapes, and barley [18]. In resistant and susceptible cultivars, DRFL1a zinc-finger transcription factors showed increased expression. Genes related to transcription factors bZIP and bZip type bZIP1 have been reduced in both susceptible and resistant cultivars. One type of autotrophic organism is plants, which produce their carbon skeletons through the process of photosynthesis in the form of sugars. These carbon skeletons are necessary as structural components and energy sources for plant growth and development. Similar to many other organisms, plants respond to carbon oscillations caused by changes in photosynthetic efficiency or metabolic status, and their growth and development are regulated accordingly [14]. Plants can sense various sugars, including sucrose, hexose, trehalose, and some exogenous responses from specific the type of sugar [53]. Expression of several members of the STP family (sugar transporter protein), including STP1, STP4, STP13, and STP14, is strongly suppressed by sugars, and STP1 is one of the suppressed genes as demonstrated by genome-wide analysis, and it is one of the repressor genes as shown by genome analysis [6]. In the research, sugar transporter protein (STP) has been identified as a key and important hub gene in resistant cultivars and was Score 10 based on the MCC method. PPi is produced as a by-product of several biosynthetic processes for macromolecules, including protein, RNA and cellulose. This proton pump Especially coexists with H-PPase in the single cell membrane vacuole in plant cells [48]. This property is related to the physiological function of H-Pase in plant cells. According to the first and second properties, H-Pase is a basic model for studying the mechanism of hydrolysis of a high-energy phosphate bond and the proton displacement. This gene actions in the molecular functional group. In studies, vacuolar H + -pyrophosphatase has been introduced as an important hub gene with a score of 48 based on the MCC method and rank 1 in susceptible cultivars. Based on the results of meta-analysis as well as qPCR, *TaSAG7*, and *PEPC* genes are effective in responding to drought stress. The expression behavior of *PEPC* gene was reversed based on the prediction of meta-analysis in susceptible and resistant cultivars, so that in susceptible cultivars under drought stress there was a decrease and in resistant cultivars under drought stress a significant increase was observed compared to the control. This expression behavior was investigated in the laboratory by qPCR in drought tolerant and sensitive wheat cultivars and confirmed the results of meta-analysis. As a result, this gene can be considered as one of the distinguishing genes of resistant and susceptible wheat cultivars and it can be used to study and screen for resistance and drought sensitivity of different. The expression behavior of *TaSAG7* gene based on meta-analysis prediction has increased in drought tolerant cultivars. This expression behavior was investigated in the laboratory by qPCR in drought tolerant and susceptible wheat cultivars and the results of meta-analysis were confirmed. As a result, this gene has been introduced as one of the distinguishing genes between drought tolerant and susceptible wheat cultivars.

## Conclusion

Finding markers to identify susceptible and resistant cultivars in crops can be of particular importance in breeding programs. In this study, using meta-analysis, we found genes that able to show contradictory behavior in resistant and sensitive cultivars and revealed some drought-responsive genes. expectantly, with the development of this method, these genes will be used to detect resistance cultivars to specific environmental conditions.

## Supplementary Information

**Additional file 1: ** Sensitive RMA.

**Additional file 2: **Tolerance RMA.

## Data Availability

All initial data is taken from the NCBI GEO (https://www.ncbi.nlm.nih.gov/geo/). The results are all available and will be provided as a supplementary file if needed.

## Abbreviations

Pn: Net photosynthetic rate
GO: Gene Ontology
RWC: Relative water content
